# P05-01 Change in cardiorespiratory fitness in midlife and incident hypertension

**DOI:** 10.1093/eurpub/ckac095.068

**Published:** 2022-08-29

**Authors:** Tobias Holmlund, Björn Ekblom, Elin Ekblom-Bak

**Affiliations:** The Swedish School of Sport and Health Sciences, GIH, Åstrand Laboratory of Work Physiology., Stockholm, Sweden

**Keywords:** Hypertension, cardiorespiratory, fitness, change, risk

## Abstract

**Background:**

Low cardiorespiratory fitness (CRF) is associated with higher blood pressure and risk of incident hypertension. However, existing literature has mainly investigated CRF at baseline. For HEPA initiatives, it is important to know how change in CRF in midlife associate with incident hypertension, and whether this varies between sexes, age and baseline CRF.

**Methods:**

91,728 individuals (20-79 years, 48% women, free from hypertension at baseline) from the Swedish workforce who had completed two health profile assessments in a nationwide occupational health service screening between 1986 and 2019 were included. CRF (assessed as VO2max) was estimated using a submaximal cycle test. Change in CRF between the two tests was expressed as % change in absolute CRF (L·min-1) per year and categorized as “maintainers” (-1% to 1% change/year), “decreases” (≥-1% change/year) or “increasers” (≥1% change/year). Incident hypertension was defined as having a blood pressure >140/90 mmHg or diagnosed with hypertension at the second test. Binary logistic regression was used to assess OR (95% CI) for hypertension at the second test between maintainers, decreases and increasers. All analyses were adjusted for sex, age, heart medication, time between tests, education, and change in other lifestyle variables between the two tests (stress, diet, exercise, smoking).

**Results:**

Compared to maintainers (set as reference), OR for decreases was 1.27 (1.18-1.38) and increasers OR 0.99 (0.89-1.09). Isolating midlife participants (40-60 years), OR for decreasers was 1.47 (1.30-1.66) and increasers OR 0.75 (0.64-0.86). Looking at sex differences, male decreasers had OR 1.24 (1.12-1.36) and female decreasers OR 1.36 (1.20-1.56), compared to maintainers, while OR for male and female increasers was 0.93 (0.83-1.03) and 0.97 (0.83-1.13). For participants with low CRF at baseline (<32·kg-1·min-1), OR for decreasers was 1.70 (1.51-1.92) and increasers 1.32 (1.18-1.49). Corresponding ORs in those with high CRF at baseline were 0.90 (0.80-1.02) and 0.43 (0.26-0.71).

**Conclusions:**

Decrease in CRF with >1% per year associated with significant higher risk for incident hypertension, while maintaining or increasing CRF had similar risk associations. This was seen in both men and women, different age-groups and baseline level of CRF. Health enhancing promotion strategies to maintain or increase CRF level is highly clinically relevant.

